# A comprehensive bibliographic study on mental illness

**DOI:** 10.1017/neu.2024.64

**Published:** 2025-01-30

**Authors:** Yuanzhao Ding, Shan Chen

**Affiliations:** 1 Social Sciences Division, University of Oxford, Oxford, UK; 2 Science of Learning in Education Centre, National Institute of Education, Nanyang Technological University, Singapore

**Keywords:** Mental illness, psychological disorders, bibliographic study, Web of Science, mental health research

## Abstract

This study presents a comprehensive analysis of recent mental illness research by utilizing an advanced bibliographic method capable of analyzing up to 12,965 papers indexed in the Web of Science database, overcoming the limitations of traditional tools like VOSviewer, which typically analyze fewer than 1,000 papers. By examining a vast dataset, this study identifies key trends, significant keywords, and prominent contributors, including leading researchers, universities, and countries/regions, in the field of mental illness research. Additionally, the study highlights eight major contributors to mental health problems, offering critical insights into the field’s current state. The findings underscore the importance of advanced bibliographic methods in providing a more detailed and accurate overview of mental illness research. This analysis not only enhances the understanding of young scholars entering the field but also uncovers significant trends and identifies notable gaps in the literature. The study advocates for continued innovation and interdisciplinary collaboration to deepen understanding and address unresolved challenges in mental health research.

## Graphical abstract

**Figure:**
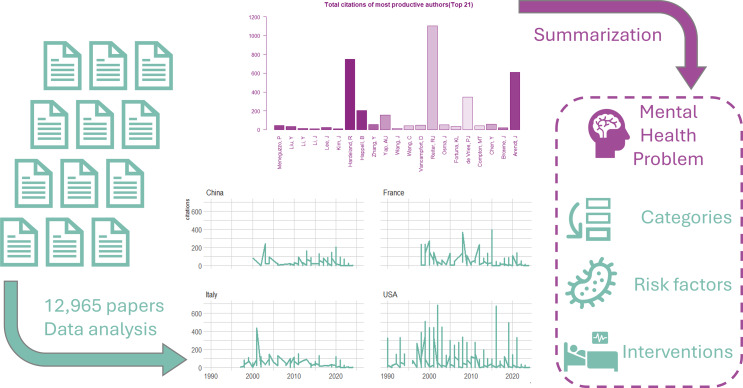

Significant outcomes:

1. **Enhanced Bibliographic Analysis:** The novel method applied in this study surpasses traditional approaches by allowing the simultaneous analysis of 12,965 papers, offering a more comprehensive and accurate overview of mental illness research.
2. **Discovery of Prevailing Research Trends:** The study identifies critical keywords and thematic areas in recent mental illness research, outlining current trends and key focuses in the field.
3. **Addressing Research Gaps:** Significant gaps in the literature are revealed, especially in underexplored areas of mental health, emphasizing the need for further investigation and innovation.

Limitations:

1. **Database Dependency:** The study relies solely on the Web of Science database, which may limit the comprehensiveness of the analysis by excluding relevant papers from other databases.
2. **Keyword Selection Bias:** The analysis depends on predefined keywords, which may introduce bias by potentially missing relevant papers that use different terminologies for mental illness.
3. **Static Nature of Data:** The data is based on a snapshot of publications at a specific time, limiting the ability to capture evolving trends and emerging research topics in real-time.

## Introduction

Mental illness is a crucial topic in psychology, garnering substantial attention from both researchers and practitioners (Richards and Stenner, 2022). This paper adopts an innovative bibliographic study method that enhances traditional approaches like VOSviewer (Huang *et al*., 2022). While traditional VOSviewer typically analyzes up to around 1,000 papers (Chen and Ding 2023a), our method accommodates the analysis of up to 12,965 papers simultaneously (Table 1). This advancement allows for the capture of more relevant information and minimises errors, resulting in a more accurate and comprehensive examination of recent publications on mental illness indexed in the Web of Science database (Pranckutė, 2021).

**Table 1.** Comparison between traditional bibliography (using VOSviewer as an example) and method used in this study

	VOSviewer	Method used in this study
Number of papers analysed	Mostly up to 1000 papers	>10’000 papers
Software used	VOSviewer	R package
Advantages	Simple and mature method	Comprehensive method highlighting word importance, connections, and correlations across numerous studies.
Disadvantages	Difficult to show trends over time	Complex process involving R coding
References	(Chen and Ding 2023b, Chen and Ding, 2024)	

The study systematically identifies and examines key papers published in the field of mental health, focusing on the most influential research in recent years. By employing this advanced bibliographic method (Echchakoui, 2020), the research successfully uncovers the dominant keywords associated with mental illness studies, highlighting the most critical themes that are currently shaping the field. These keywords serve as indicators of prevailing research trends and areas of focus within the broader context of mental health (Hernández-Torrano *et al*., 2020).

In addition to identifying significant keywords, the study also analyzes the contributions of leading researchers, universities, and countries/regions (Ding and Yang 2022). This analysis reveals the primary contributors to the field, offering insights into the global distribution of mental illness research (Vigo *et al*., 2022). The findings indicate that certain universities and research institutions are at the forefront of this area, producing a substantial portion of the literature (Shalaby and Agyapong, 2020). Similarly, specific countries and regions are identified as major hubs for mental health research, reflecting the geographical concentration of expertise and resources dedicated to studying mental illness (Bemme and Kirmayer, 2020).

The paper provides a comprehensive overview of the current state of mental health research, covering the progress made in understanding various mental health disorders, such as depression, anxiety, schizophrenia, and bipolar disorder. It also highlights the methodological advancements used to study these conditions. The paper identifies key gaps in the existing literature, emphasising areas where further research is needed. While significant progress has been made in understanding some disorders, others remain underexplored, necessitating more focused investigation. Additionally, the influence of major research contributors on different mental health disorders is discussed, offering a broader perspective on their impact in the field.

Additionally, the study provides a forward-looking discussion on the future directions of mental illness research. It underscores the necessity of ongoing interdisciplinary collaboration, given that mental health is a complex issue intersecting with diverse fields such as neuroscience, sociology, and public health, with particular emphasis on the integration of big data and machine learning. The paper contends that adopting innovative research approaches and methodologies is crucial for advancing our understanding of mental illness. This includes tackling the complexities and challenges involved in diagnosing, treating, and preventing these conditions effectively.

## Materials and methods

The study was conducted at the Social Science Divisions of the University of Oxford, UK, and in Singapore City. Using the Web of Science database (Singh *et al*., 2021), keywords related to mental health were searched, including terms such as ‘mental health issues’, ‘mental illness’, ‘mental health problems’, ‘mental disorders’, ‘psychological disorder’, ‘psychiatric disorder’, ‘neuropsychiatric disorder’, ‘emotional disorder’, ‘behavioral disorder’, ‘cognitive disorder’, and ‘cognitive function’.

**Figure 1.**
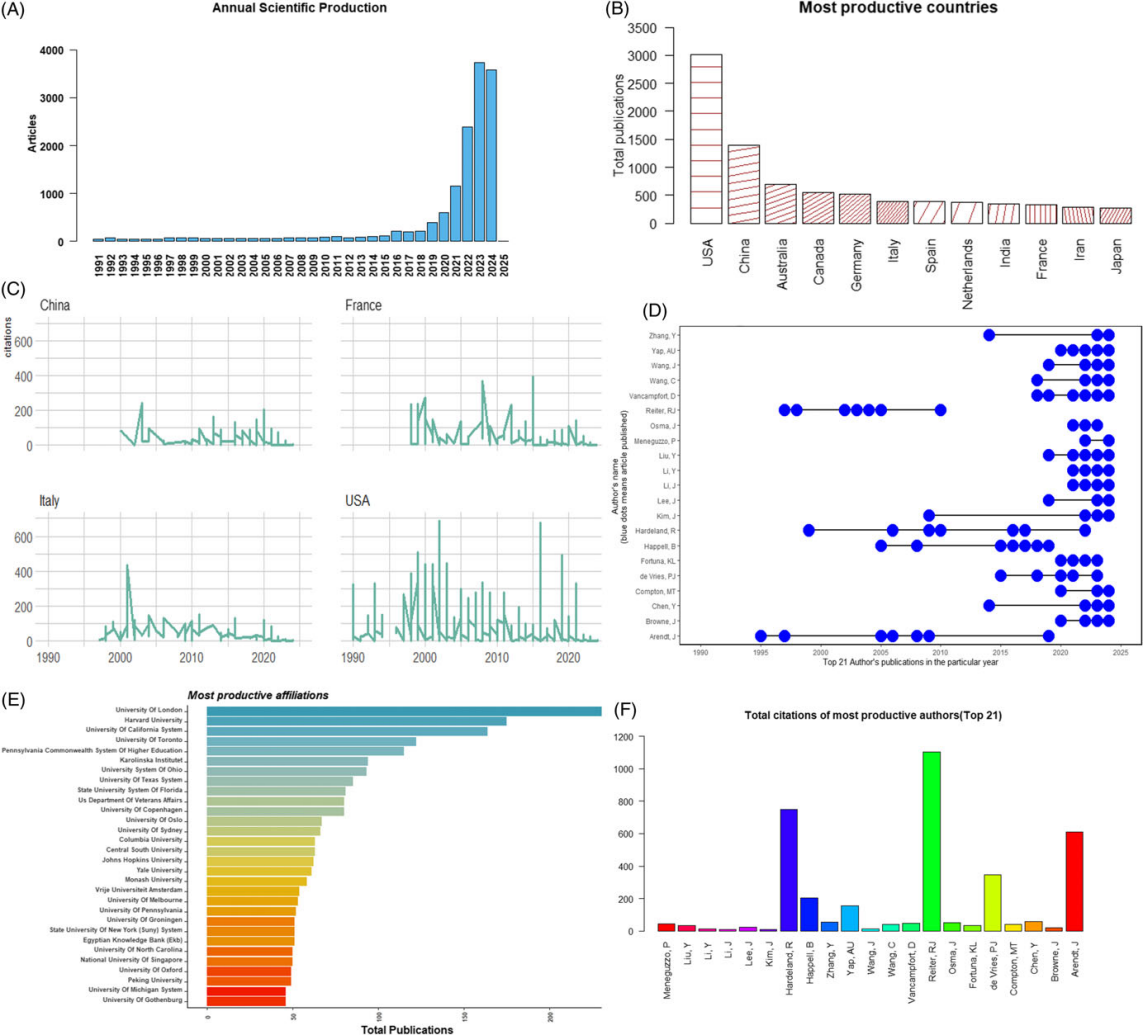
Paper productivity by year (A), country/region (B-C), author (D, F), and organisational output (E).

The topic of ‘mental health problems’ has been published for decades, and the search yielded 12,965 papers (duplicates removed), as shown in Figure 1. Data were downloaded on 6th August 2024. For the analysis of productivity by countries/regions and authors, the first author was considered the primary contributor to the articles.

## Results

We analysed global trends in the field of mental health research. As shown in Figure 1(A), there has been a significant increase in publications focused on ‘mental health issues’ from 2019 to 2024, compared to the total output prior to 2019. The number of publications reached 1,000 in 2021 and surged to over 3,000 by 2023. This indicates a substantial rise in the number of papers in this field over the past few years, reflecting growing interest and concern regarding mental health.

**Figure 2.**
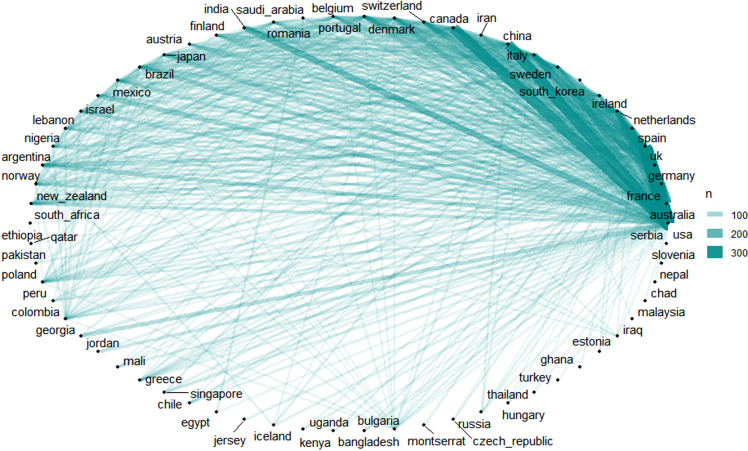
Collaboration between countries/regions, with lines representing research partnerships.

Next, we analysed the contributions of countries and regions. Figure 1(B) ranks the top 12 countries/regions that have contributed the most to mental health research. The USA leads with over 3,000 papers, followed by China, Australia, Canada, Germany, Italy, Spain, the Netherlands, India, France, Iran, and Japan. This ranking highlights the close collaboration among these nations, which has been pivotal in shaping the development of mental health research. Some countries, such as the USA, have a stronger research foundation and have been active in this field for longer, while others with larger populations and higher rates of mental health issues, like China and India, require extensive international collaboration. Figure 1(C) further shows the citation counts, revealing that publications from the USA are the most frequently cited. Figure 2 illustrates the collaborative relationships between the USA and other countries, with strong ties to France, Germany, Australia, Spain, the Netherlands, Sweden, Italy, China, Canada, and Ireland.

**Figure 3.**
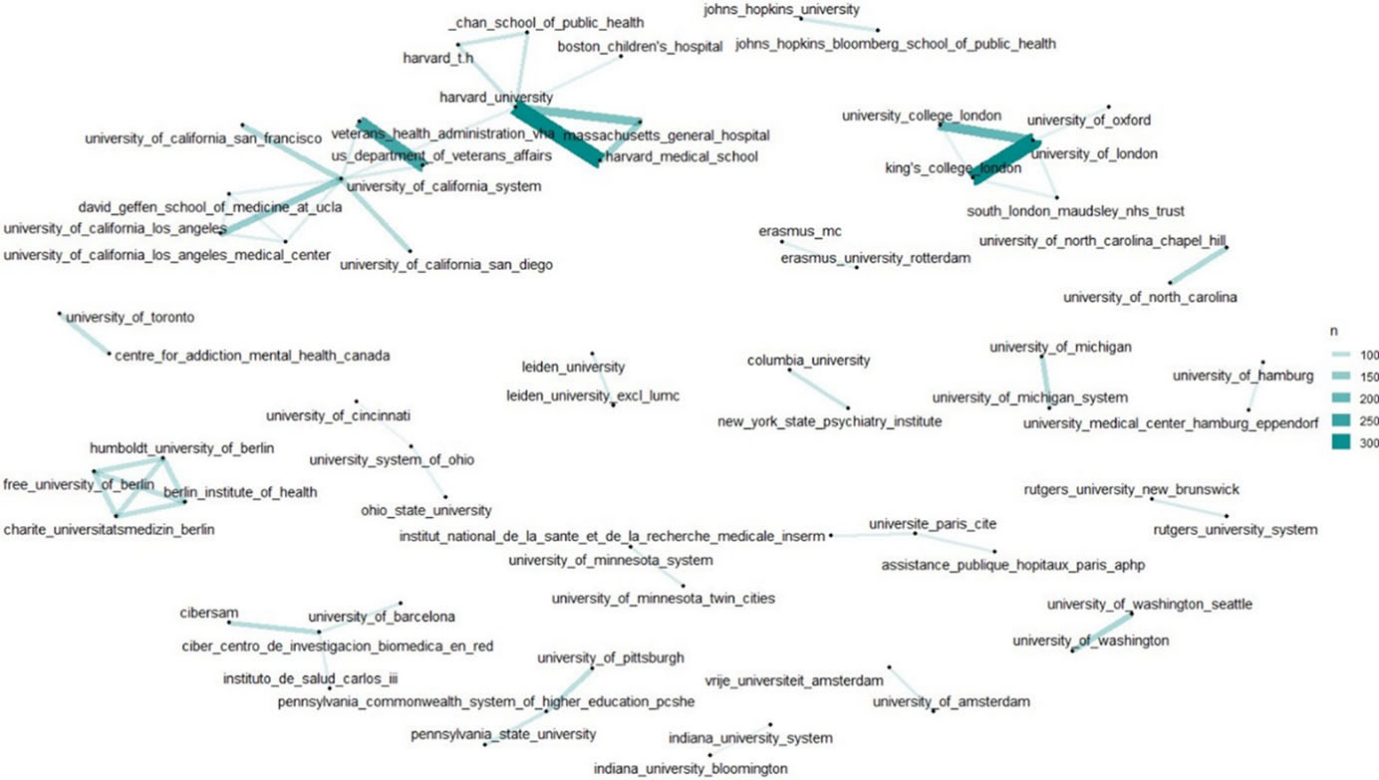
Research collaboration between organisations, indicated by connecting lines.

We also analysed the major organisations contributing to mental health research. Figure 1(E) highlights the top 30 most productive institutions in the study of mental disorders, with the University of London, Harvard University, and the University of California System standing out as particularly influential. Figure 3 illustrates the organisational collaboration network, showing that the University of Oxford collaborates with the University of London and King’s College London, while Harvard University often works with Harvard Medical School and Massachusetts General Hospital. The growing trend of collaboration, particularly international partnerships, is evident in recent years, driving progress in the field of mental health research.

**Figure 4.**
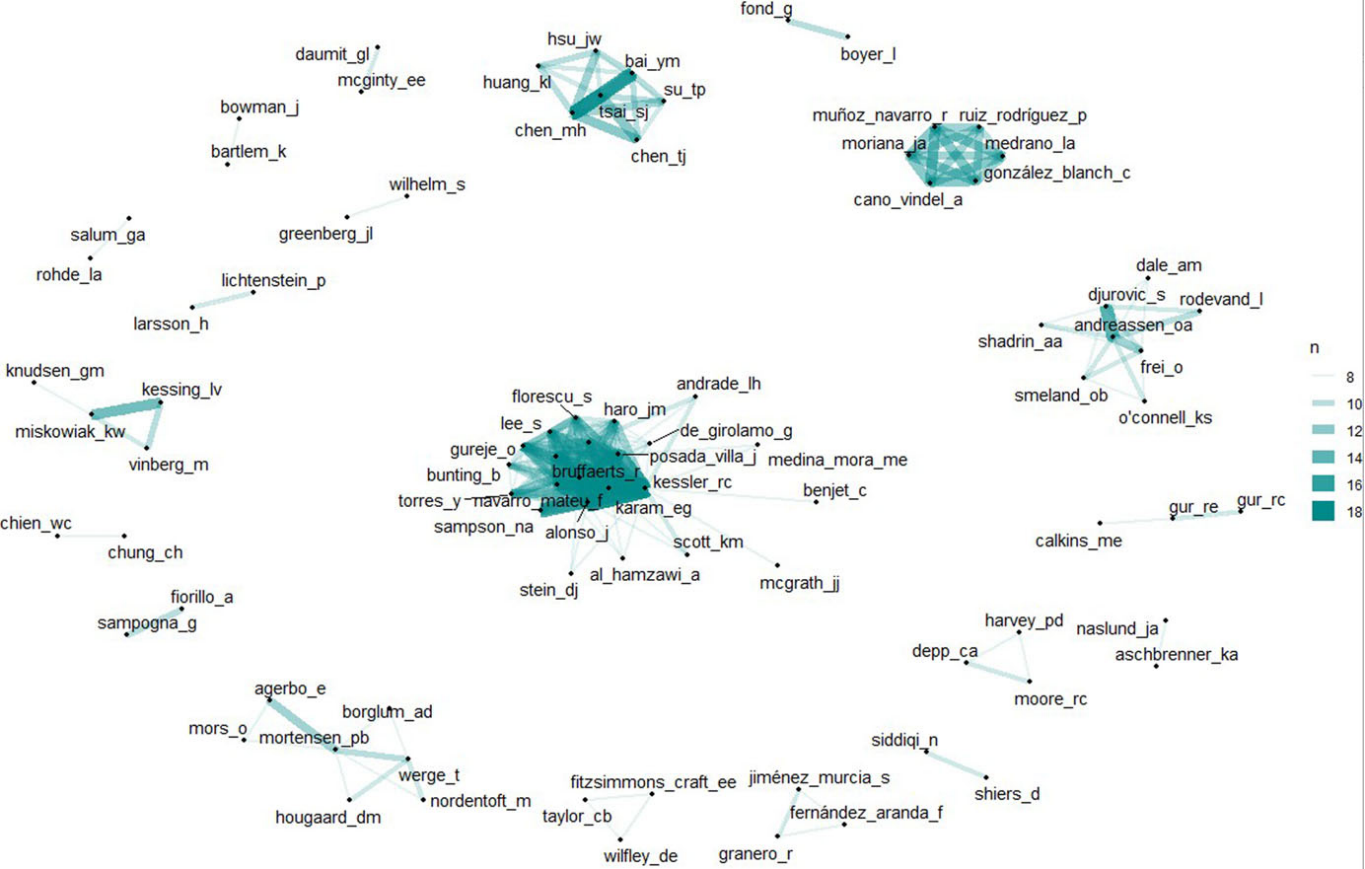
Co-authorship network, with lines representing research collaboration.

Finally, we examined the most influential authors in mental health research. As Figure 1 (D) displayed, the most productive authors for whom have been published many papers from the year 1990 to August 2024, as counted in the contribution of first author. The most influential author is presented in Figure 1(F), Reiter, R J is the most influential author in mental health area, whose work is most frequently cited by other researchers. He is a prominent expert in biology known for his work exploring the relationship between biological mechanism and well-being mental health. He particularly concentrates on circadian rhythms, melatonin, chronobiology, and antioxidant properties in affecting physical and mental changes, regulating physical and mental health. For instance, he’s research team proposed that how chronobiology adjusting biology rhythms to cause mental health disorders (Sainz *et al*., 2003, Hardeland *et al*., 2009, Cipolla-Neto *et al*., 2014). A teamwork also found that melatonin regulating biological rhythms and metabolism that speed up the apoptotic death of cancer cells (Sainz *et al*., 2003). Hardeland, R and Arendt, J and de Vries, P J are also well-known in mental health research area, and they won hundreds of citations. Hardeland, R mainly focus on the effect of melatonin, He extensively examine the neuroprotective and immunomodulatory effects in protecting nerve cells, regulating sleep disorders, and metabolic syndrome to lower the risk of neurologic disease (including mood disorders, depression) due to the damage of cell recycling system brings fatal nerve disease (Hardeland, 2018). Arendt, J and de Vries, P J also concentrate on their work on circadian rhythms and sleep disorders to minimise the side effects and reveal the underlying mechanisms of sleep and mental health problems. One famous study from *The Lancet Psychiatry* reported that sleep difficulties mediating paranoia and hallucinations, and it also contribute to explain the occurrence of mental disorders (Freeman *et al*., 2017). As a systematic meta-analysis study with 72 interventions (*N* = 8608 participants) summarised that improving sleep quality led to a greater improvements in mental health (Scott *et al*., 2021). Other studies also reported sleep disorders in 2019 novel coronavirus (COVID-19) disease outbreak (Pinto *et al*., 2020), and Zhang *et al*., (2017) found unhealthy sleep patterns increase the risk of mental health disorders. The authors listed above, who are the most influential, are also the most productive, having published numerous papers over the past decades. Figure 4 illustrates the co-authorship network, showing the collaborative relationships between these authors. For example, Bruffaerts R frequently collaborates with Kessler R C, Alonso J, Karam E G, and Sampson N A, as evidenced by the strong and tightly connected lines representing their social network ties.

**Figure 5.**
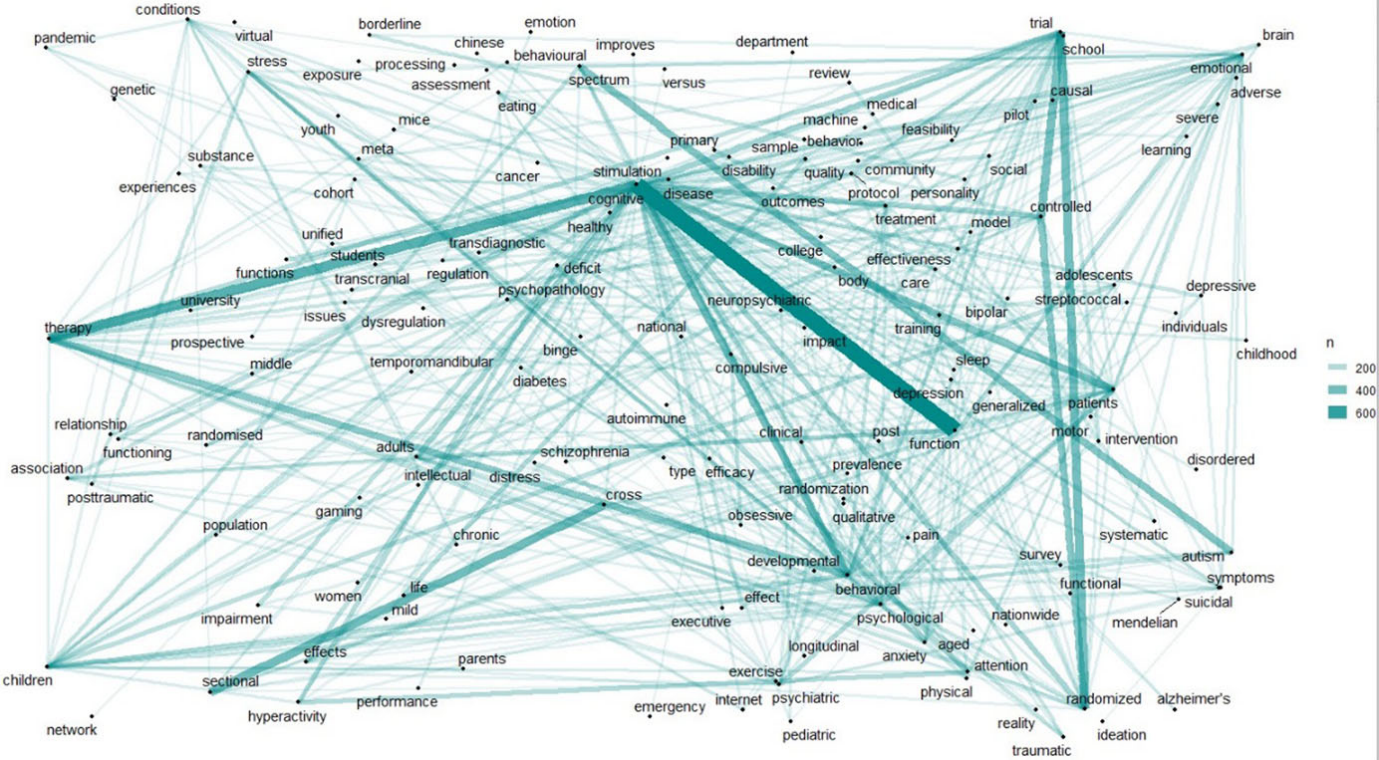
Title name map, with lines representing connections between words.

Keywords from titles were extracted to conduct a bibliographic analysis. As seen in Figure 5, existing research in the mental health area mainly focused on cognitive function, cognitive therapy, cross-sectional studies, randomised trials, autism spectrum, cognitive behavioural therapy, cognitive trials, treatment, and intervention, among others. Mental health problems were strongly related to cognitive function, cognitive therapy, behavioural therapy, attention deficit hyperactivity disorder (ADHD), quality of life, psychiatric patients, children, adolescents, students, anxiety, cognitive impairment, post-traumatic stress disorder (PTSD), systematic reviews, chronic conditions, psychological symptoms, depressive patients, bipolar disorder, cognitive training or pilot studies, questionnaires, scales, Alzheimer’s disease, therapy effectiveness, paediatric autoimmune conditions, internet gaming, post-streptococcal autoimmune disorders, schizophrenia spectrum, and causal inference, among others.

**Figure 6.**
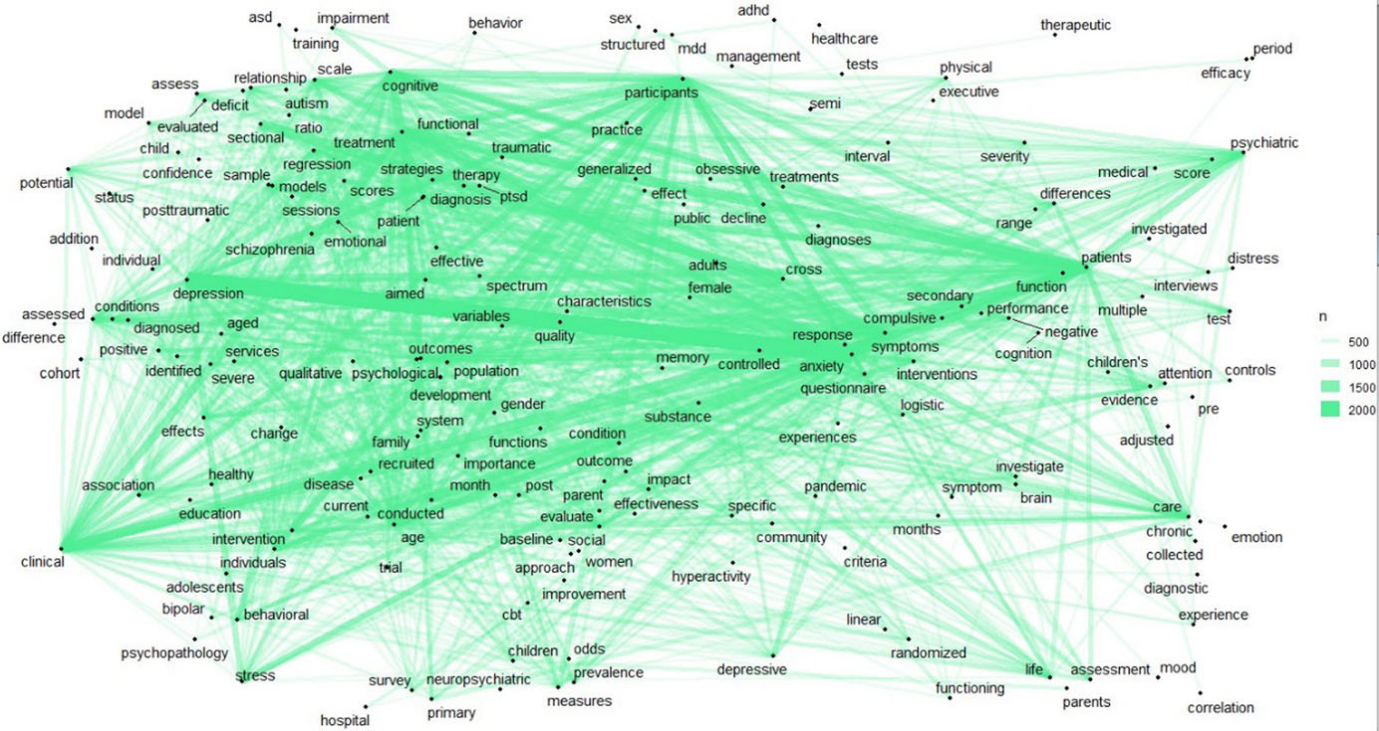
Abstract map, with lines representing connections between terms.

Figure 6 displayed the keywords extracted from abstracts, which primarily included terms such as depression and anxiety, cross-sectional studies, patient treatment, clinical treatment, cognitive function, quality of life, cognitive effects and impairment, ADHD, bipolar disorder and schizophrenia, PTSD, intervention effects, regression relationships, brain studies, treatment effectiveness, and individual differences. Many of these keywords pertained to information about study subjects, such as participants, patients, parents, children, females, adolescents, and families. Other keywords focused on research methods, including terms like medical, psychiatric, investigated, questionnaire, measures, assessment, and interview. Additionally, several keywords centred on psychological issues themselves, such as depression and stress. Figure 6 also illustrated the connections between these keywords, demonstrating how they interrelated within the context of mental health research.

**Figure 7.**
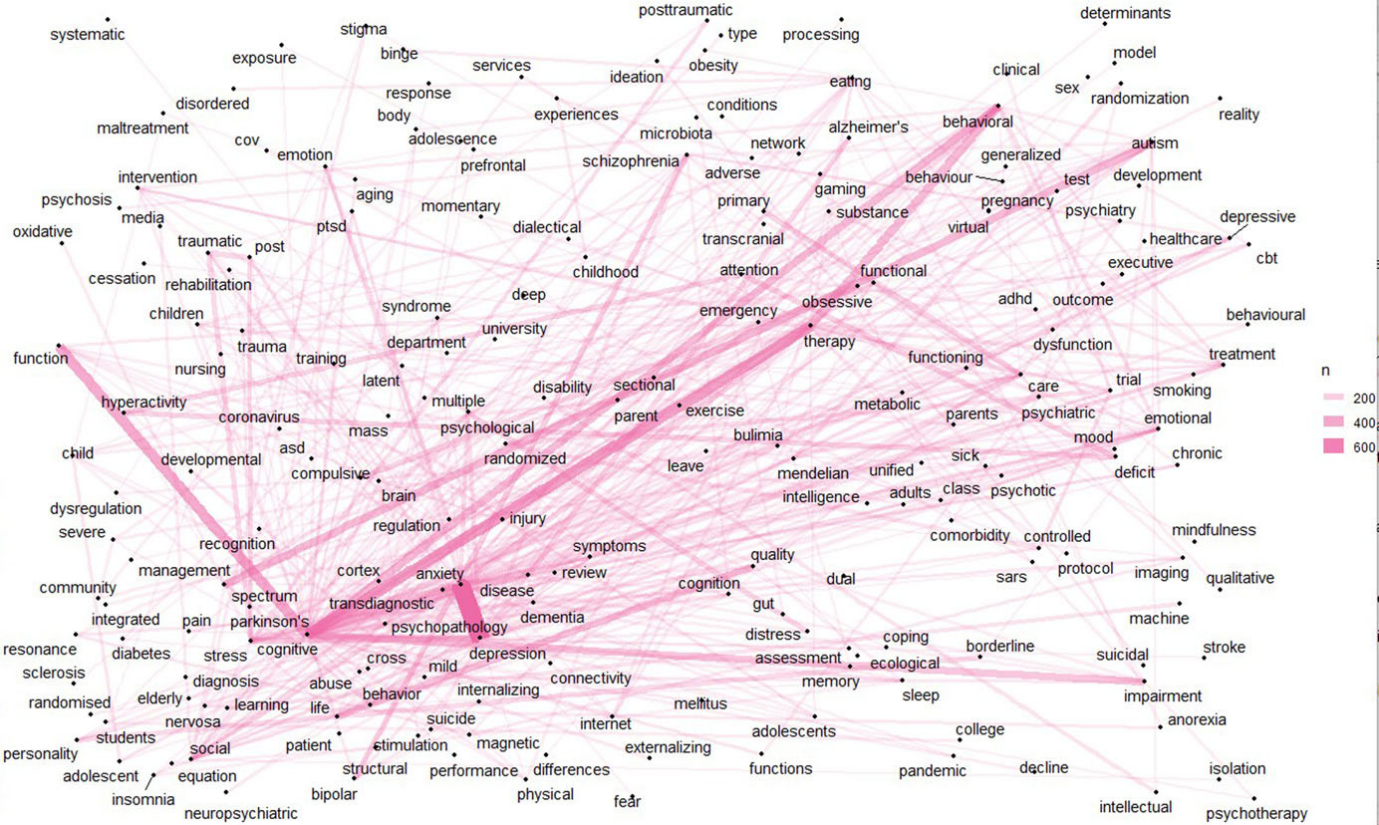
Keywords network map, with lines showing connections between terms.

Figure 7 collected the keywords of articles, which includes anxiety depression, cognitive therapy, autism spectrum, life quality, cognitive impairment, bipolar and schizophrenia, ADHD, obsessive compulsive, psychological distress, Alzheimer’s disease, PTSD, cognitive disease, machine learning, children and adolescents, magnetic resonance imaging, brain cognitive, emotional regulation, nervosa anorexia, cognitive dysfunction, chronic disease, social stigma, suicidal ideation, sleep cognitive, anxiety sleep, oxidative stress, personality psychopathology, eating disorders, diabetes type, CBT cognition, psychiatric comorbidity, eating nervosa, internet gaming, and etc.

**Figure 8.**
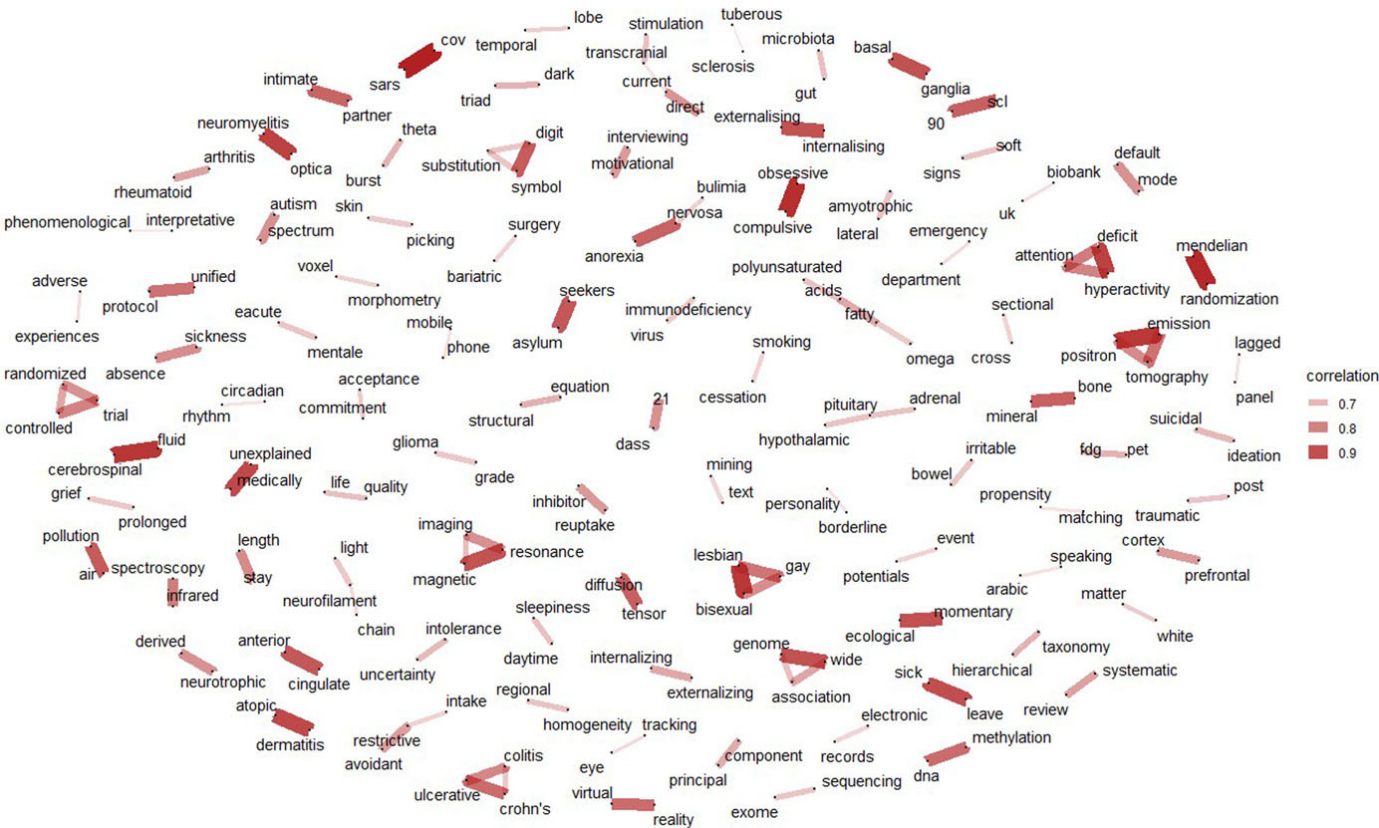
Keywords correlations map, with lines showing the connections between words.

Figure 8 presents the correlations among the keywords collected from the articles, revealing various connections that enhance our understanding of mental health disorders. Among the noteworthy links are those between ’sick’ and ‘leave’, which may indicate the relationship between mental health issues and work absence, highlighting the impact of mental illness on employment and productivity. Another significant link is between ‘DNA’ and ‘methylation’, reflecting the growing interest in the genetic and epigenetic factors associated with mental health disorders. This connection points to research exploring how molecular changes, such as DNA methylation, play a role in the onset and progression of conditions like depression and anxiety. These connections, among others highlighted in Figure 8, provide valuable insights into the complex interplay of factors involved in mental health, offering new avenues for research and potential interventions.

In a nutshell, a comprehensive bibliography contained the summarised features of publication productivity, time span, country/region, affiliation, co-authorship, authors’ contributions, citations, and so on. It provided a concise overview of the resources and key points within the field of mental health disorders, which benefited conducting an in-depth analysis of this topic.

## Discussion

### Categories of mental health problems

Overall, mental health problems can be categorised into externalising and internalising disorders to signify behavioural and emotional issues (Singh *et al*., 2021). Externalizing disorders are characterised by observable and manifest behaviours. These behaviours involve compulsive, repetitive, and aggressive impulses, including misbehaviour and delinquency (e.g., theft, deception, offensive actions, attention deficit, hyperactivity, and impulsivity). Such behaviours create difficulties for individuals in social and academic environments, as well as challenges in friendships and intimate relationships.

Internalizing disorders, on the other hand, are characterised by internal emotional distress and discomfort (Lee *et al*., 2021). These disorders are less visible and can be difficult to recognise and articulate. For example, depression, also known as distressed symptoms, involves a loss of interest in activities, persistent sadness, and feelings of worthlessness or hopelessness. Anxiety is marked by feelings of discomfort and nervousness, ranging from mild to severe worries, desires, and fears. Often, these feelings are persistent and unavoidable. Stress refers to mental strain and tension that prompts individuals to respond to upcoming challenges and threats. Obsessive-compulsive disorder involves involuntary recurring thoughts (obsessions) and actions (compulsions) that compel individuals to perform certain activities or dwell on intrusive ideas.

### Contributors to mental health problems

To explain the reasons for mental disorders, classification is useful in identifying potential factors. This can be restricted to the following aspects, though it is not limited to them: firstly, genetic factors (Arango *et al*., 2021). Genetic predispositions, often indicated by a family history of mental illness, can impart specific traits to offspring, making them more likely to develop similar symptoms. For example, individuals with inherited genetic variations may exhibit a tendency toward negative feelings or emotions, such as anxiety, stress, distress, or bipolar disorder. A relevant case study is the identification of the COMT gene, which has been linked to various mental disorders, including schizophrenia and bipolar disorder. In addition, epigenetic mechanisms can trigger chromosome mutations due to environmental variations, which describe the mutations of heritable traits without changes in the DNA sequence (Berger *et al*., 2009). Epigenetic regulation impacts the nervous system and proteins via altered transcription and translation processes, leading to mental hindrances or disorders (e.g., autism spectrum disorder, schizophrenia, drug addiction) due to abnormalities in chromatin remodelling (Tsankova *et al*., 2007, Chmielewska *et al*., 2019, Miao *et al*., 2020). Miao *et al*., (2020) explored the role of epigenetic functions in mental illnesses such as impaired social interactions, memory issues, depression, and stress symptoms. The interaction between genetic factors and environmental influences, as shown in epigenetic research, further emphasises the need to examine genetic predispositions in the development of mental disorders.

Secondly, biological factors (Arango *et al*., 2021). Mental disorders are closely related to the chemical reactions occurring in the brain. Instabilities in neurotransmitters – such as dopamine and serotonin – are strongly linked to the development of mental disorders. For example, insufficient levels of dopamine and serotonin are associated with major depressive symptoms. In addition to neurotransmitter imbalances, other biological factors such as hormonal disruptions, traumatic brain injuries, developmental delays, and brain infections can also contribute to mental health conditions. Hallucinations and illusions are classic examples that illustrate the complex interactions between biological factors and mental health, further emphasising the need for biological interventions in treating such conditions.

Thirdly, psychological causes (Da Silva *et al*., 2020). Mindsets that focus excessively on negative aspects of life, adherence to false beliefs or outdated ideologies, and a rejection of science and education can lead to mental health challenges. Psychological factors such as these can contribute to a range of mental disorders, including anxiety, bipolar disorder, stress, PTSD, and depression. The development of these conditions can often be traced to cognitive patterns that reinforce negative thought processes and dysfunctional behaviour, which can exacerbate emotional distress and impair coping mechanisms.

Fourthly, environmental influences (Sameroff and Seifer 2021). Personal experiences, particularly adversities, traumatic events, abuse, maltreatment, lack of emotional support, and low socio-economic status, can create an environment that is conducive to mental illness. Economic inequalities, job insecurity, and limited job opportunities further compound these environmental stressors, potentially contributing to the development of mental disorders. The broader societal context in which individuals live, including exposure to poverty and violence, often shapes their mental well-being and can lead to long-term psychological distress.

Fifthly, living habits and customs can also contribute to mental disorders (Blom *et al*., 2021). Habits such as substance abuse, poor diet, malnutrition, and obesity can lead to emotional distress, anxiety, and eating disorders. These lifestyle factors can affect an individual’s physical health, which in turn has a significant impact on their mental health. For example, poor nutritional habits can contribute to mood fluctuations and cognitive impairments, while substance abuse can cause addiction-related mental health issues. Therefore, it is crucial to address these lifestyle factors as part of mental health prevention and treatment strategies.

Sixthly, physical health issues (Kivimäki *et al*., 2020). Individuals with chronic diseases such as heart disease, diabetes, hypertension, long-lasting pain, and cancer may experience increased stress, which can exacerbate mental health issues. Furthermore, the side effects of medications used to treat physical health conditions can also contribute to mental distress. The interplay between physical health and mental well-being is complex, as individuals dealing with chronic illnesses may experience heightened vulnerability to anxiety, depression, and other mental health conditions, making integrated care essential for these patients.

Seventhly, accidental occurrences (Melo *et al*., 2022). Significant life events and transitions, such as divorce, car accidents, the loss of loved ones, and adapting to new challenges, can have profound effects on mental health. These events often lead to emotional distress, grief, and sometimes PTSD. The psychological impact of these occurrences can vary depending on the individual’s resilience and support system, but they are undoubtedly a contributing factor to the development of mental health conditions.

Eighthly, socio-cultural constraints (Kiselev *et al*., 2020). Stigma, bias, and discrimination, as well as societal expectations, social regulations, traditions, cultural conventions, and norms, can all have significant effects on an individual’s mental well-being. The pressure to conform to societal ideals can lead to feelings of inadequacy, anxiety, and depression, particularly when individuals feel marginalised or excluded. These socio-cultural factors can create environments where mental illness is exacerbated or underrecognized, highlighting the need for societal change to support mental health awareness and acceptance.

### Interventions of mental health problems

Physical therapy, psychotherapy, and chemotherapy are widely recognised as effective methods for treating mental health disorders, each contributing in distinct ways to the intervention and management of mental illnesses (Smith *et al*., 2020). Physical therapy involves a variety of practices aimed at enhancing physical well-being, which can in turn improve mental health. These practices include yoga, exercise, massage, electrotherapy, hydrotherapy, cryotherapy, thermal therapy, therapeutic ultrasound, mindfulness, meditation, and social activities. Additional therapies such as art and music therapy, acupuncture, herbal remedies, and nutritional guidance also play a role in helping to strengthen muscles, reduce pain, and promote overall physical and mental health.

Psychotherapy, on the other hand, consists of several therapeutic approaches that target emotional and behavioural issues (Cuijpers *et al*., 2016). Cognitive behavioural therapy (CBT) is one of the most common forms, helping individuals reframe negative thought patterns and behaviours (Nakao, Shirotsuki and Sugaya 2021). Dialectical behaviour therapy is another widely used method, focusing on emotional regulation and interpersonal effectiveness (Comtois *et al*., 2007). Psychodynamic therapy delves into the unconscious mind, promoting self-awareness and understanding of deeper emotional issues, while humanistic therapy emphasises self-fulfilment and personal growth, helping individuals explore and achieve their fullest potential (Leichsenring, Luyten *et al*., 2015).

Chemotherapy, in the context of mental health treatment, refers to the use of medications to manage a range of psychiatric conditions (Zare *et al*., 2019). Antidepressants, antipsychotics, anxiolytics, and mood stabilisers are commonly prescribed to treat disorders such as depression, anxiety, schizophrenia, and bipolar disorder. Alongside these medications, medical devices like transcranial magnetic stimulation (Kan *et al*., 2023) and electroconvulsive therapy (Watts *et al*., 2021) are also utilised to provide relief for more severe or treatment-resistant conditions.

In summary, mental health disorders are generally categorised into externalising and internalising types, each influenced by a variety of internal and external factors. Understanding these contributing elements is crucial for designing effective treatment strategies and ensuring successful intervention outcomes. Through a combination of physical, psychological, and medical interventions, individuals suffering from mental health disorders can find the support they need for recovery and well-being.

### Limitations to solutions: big data and machine learning on mental health

The analysis conducted highlights the intricate complexity of mental illness, emphasising the need for innovative approaches to enhance our understanding and treatment of these conditions. A thorough review of the literature reveals several limitations in the current field of mental health research. These limitations include: (1) the lack of systematic summaries of texts and data, (2) the occasional overemphasis on specific discoveries related to mental illness, and (3) the reliance on the subjective experience of mental health professionals, which can introduce unavoidable human error into diagnoses and treatment plans (Bradford *et al*., 2024).

Addressing these limitations, this study introduces a novel perspective on the potential of big data and machine learning in advancing mental health research (Al Banna *et al*., 2023). The application of these technologies, which have already shown substantial benefits in fields such as facial recognition (Al Banna *et al*., 2023), autonomous driving (Bachute and Subhedar, 2021), and species distribution prediction (Chen and Ding, 2022), presents a transformative opportunity for mental health.

Firstly, the establishment of a comprehensive database incorporating diverse human information – such as age, education, family background, dietary habits, physical measurements, and psychological conditions – could provide a robust resource for mental health research. This extensive dataset would enable researchers to capture the multifaceted nature of mental disorders and better understand the complex interplay of various contributing factors.

Utilising machine learning models, such as neural networks, on this enriched dataset could significantly enhance predictive accuracy for mental health conditions (Su *et al*., 2020). Machine learning algorithms have the capability to identify patterns and correlations that traditional research methods might overlook. By training these models with large-scale data, researchers can develop more precise tools for predicting mental health issues and identifying individuals at risk, thereby enabling earlier and more targeted interventions.

Moreover, the integration of machine learning into mental health research facilitates the development of early intervention strategies (Chekroud *et al*., 2021). Predictive models could forecast the onset of mental health disorders before they fully manifest, allowing for timely and personalised treatment plans. This proactive approach has the potential to substantially improve patient outcomes by addressing issues at an early stage, thus reducing the severity of mental health disorders.

The potential of big data and machine learning in mental health research is profound (Koppe *et al*., 2021). These technologies offer the capacity to process and analyse vast amounts of data, uncovering insights that can drive innovative treatment solutions and deepen our understanding of mental illnesses. As the field of mental health research continues to evolve, the application of these technologies promises to revolutionise our approach by providing more accurate diagnoses, personalised treatment options, and ultimately, enhanced mental health outcomes.

## Conclusion

In conclusion, mental illness remains a critical area of academic research, and this study has utilised the latest bibliographic methods to explore this field comprehensively. Through our analysis, we identified the primary mental disorders and the predominant research methodologies within this domain. The study highlights key trends and influential figures, providing a clear picture of the current landscape of mental health research. Furthermore, the discussion points to promising future directions, emphasising the significant potential of big data and machine learning technologies. These advanced tools offer the capability to uncover deeper insights and enhance the precision of mental health research, ultimately leading to more effective interventions and treatment strategies. By leveraging these technological advancements, researchers can address existing gaps, explore new avenues of inquiry, and contribute to a more nuanced understanding of mental illnesses. This approach not only enriches the field but also holds the promise of improving mental health outcomes on a broader scale.

## Data Availability

Data supporting this study are available from the corresponding author.

## References

[ref1] Al Banna MH , Ghosh T , Nahian MJAl , Kaiser MS , Mahmud M , Taher KA , Hossain MS and Andersson K (2023) A hybrid deep learning model to predict the impact of COVID-19 on mental health from social media big data. IEEE Access 11, 77009–77022.

[ref2] Arango C , Dragioti E , Solmi M , Cortese S , Domschke K , Murray RM , Jones PB , Uher R , Carvalho AF and Reichenberg A (2021) Risk and protective factors for mental disorders beyond genetics: an evidence-based atlas. World Psychiatry 20(3), 417–436. 34505386 10.1002/wps.20894 PMC8429329

[ref3] Bachute MR and Subhedar JM (2021) Autonomous driving architectures: insights of machine learning and deep learning algorithms. Machine Learning with Applications 6, 100164.

[ref4] Bemme D and Kirmayer LJ (2020) Global mental health: interdisciplinary challenges for a field in motion. Transcultural Psychiatry 57, 3–18. Sage Publications Sage UK: London, England 32106797 10.1177/1363461519898035

[ref5] Berger SL , Kouzarides T , Shiekhattar R and Shilatifard A (2009) An operational definition of epigenetics. Genes & development 23(7), 781–783. 19339683 10.1101/gad.1787609 PMC3959995

[ref6] Blom V , Lönn A , Ekblom B , Kallings LV , Väisänen D , Hemmingsson E , Andersson G , Wallin P , Stenling A and Ekblom Ö. (2021) Lifestyle habits and mental health in light of the two COVID-19 pandemic waves in Sweden, 2020. International Journal of Environmental Research and Public Health 18(6), 3313. 33806951 10.3390/ijerph18063313 PMC8005136

[ref7] Bradford A , Meyer AND , Khan S , Giardina TD and Singh H (2024) Diagnostic error in mental health: a review. BMJ Quality & Safety 33(10) 663–672. 10.1136/bmjqs-2023-016996 PMC11503128 38575311

[ref8] Chekroud AM , Bondar J , Delgadillo J , Doherty G , Wasil A , Fokkema M , Cohen Z , Belgrave D , DeRubeis R and Iniesta R (2021) The promise of machine learning in predicting treatment outcomes in psychiatry. World Psychiatry 20(2), 154–170. 34002503 10.1002/wps.20882 PMC8129866

[ref9] Chen S and Ding Y (2022) Machine learning and its applications in studying the geographical distribution of ants. Diversity 14(9), 706.

[ref10] Chen S and Ding Y (2023a) A bibliography study of shewanella oneidensis biofilm. FEMS Microbiology Ecology 99(11), fiad124. 37796898 10.1093/femsec/fiad124 PMC10630087

[ref11] Chen S and Ding Y (2023b) Tackling heavy metal pollution: evaluating governance models and frameworks. Sustainability 15(22), 15863.

[ref12] Chen S and Ding Y (2024) From bibliography to understanding: water microbiology and human health. Journal of Water and Health 22(10), 1911–1921.

[ref13] Chmielewska N , Szyndler J , Maciejak P and Płaźnik A (2019) Epigenetic mechanisms of stress and depression. Psychiatria Polska 53(6), 1413–1428. 32017826 10.12740/PP/94375

[ref14] Cipolla-Neto J , Amaral FG , Afeche SC , Tan DX and Reiter RJ (2014) Melatonin, energy metabolism, and obesity: a review. Journal of Pineal Research 56(4), 371–381. 24654916 10.1111/jpi.12137

[ref15] Comtois KA , Elwood L , Holdcraft LC , Smith WR and Simpson TL (2007) Effectiveness of dialectical behavior therapy in a community mental health center. Cognitive and Behavioral Practice 14(4), 406–414.

[ref16] Cuijpers P , Donker T , Weissman MM , Ravitz P and Cristea IA (2016) Interpersonal psychotherapy for mental health problems: a comprehensive meta-analysis. American Journal of Psychiatry 173(7), 680–687. 27032627 10.1176/appi.ajp.2015.15091141

[ref17] Da Silva AG , Baldaçara L , Cavalcante DA , Fasanella NA and Palha AP (2020) The impact of mental illness stigma on psychiatric emergencies. Frontiers in psychiatry 11, 573. 32636773 10.3389/fpsyt.2020.00573 PMC7319091

[ref18] Ding X and Yang Z (2022) Knowledge mapping of platform research: a visual analysis using VOSviewer and CiteSpace. Electronic Commerce Research, 22, 1–23

[ref19] Echchakoui S (2020) Why and how to merge Scopus and Web of Science during bibliometric analysis: the case of sales force literature from 1912 to 2019. Journal of Marketing Analytics 8, 165–184.

[ref20] Freeman D , Sheaves B , Goodwin GM , Yu L-M , Nickless A , Harrison PJ , Emsley R , Luik AI , Foster RG , Wadekar V , Hinds C , Gumley A , Jones R , Lightman S , Jones S , Bentall R , Kinderman P , Rowse G , Brugha T , Blagrove M , Gregory AM , Fleming L , Walklet E , Glazebrook C , Davies EB , Hollis C , Haddock G , John B , Coulson M , Fowler D , Pugh K , Cape J , Moseley P , Brown G , Hughes C , Obonsawin M , Coker S , Watkins E , Schwannauer M , MacMahon K , Siriwardena AN and Espie CA (2017) The effects of improving sleep on mental health (OASIS): a randomised controlled trial with mediation analysis. The Lancet Psychiatry 4(10), 749–758. 28888927 10.1016/S2215-0366(17)30328-0 PMC5614772

[ref21] Hardeland R (2018) The underrated circadian system and its contribution to neurodegeneration in mood disorders. Journal of Mental Health & Clinical Psychology 2(3) 17–22.30556062

[ref22] Hardeland R , Tan D-X and Reiter RJ (2009) Kynuramines, metabolites of melatonin and other indoles: the resurrection of an almost forgotten class of biogenic amines. Journal of Pineal Research 47(2), 109–126. 19573038 10.1111/j.1600-079X.2009.00701.x

[ref23] Hernández-Torrano D , Ibrayeva L , Sparks J , Lim N , Clementi A , Almukhambetova A , Nurtayev Y and Muratkyzy A (2020) Mental health and well-being of university students: a bibliometric mapping of the literature. Frontiers in Psychology 11, 1226. 32581976 10.3389/fpsyg.2020.01226 PMC7296142

[ref24] Huang Y-J , Cheng S , Yang F-Q and Chen C (2022) Analysis and visualization of research on resilient cities and communities based on VOSviewer. International Journal of Environmental Research and Public Health 19(12), 7068. 35742316 10.3390/ijerph19127068 PMC9223032

[ref25] Kan RLD , Padberg F , Giron CG , Lin TTZ , Zhang BBB , Brunoni AR and Kranz GS (2023) Effects of repetitive transcranial magnetic stimulation of the left dorsolateral prefrontal cortex on symptom domains in neuropsychiatric disorders: a systematic review and cross-diagnostic meta-analysis. The Lancet Psychiatry 10(4), 252–259. 36898403 10.1016/S2215-0366(23)00026-3

[ref26] Kiselev N , Pfaltz M , Haas F , Schick M , Kappen M , Sijbrandij M , De Graaff AM , Bird M , Hansen P and Ventevogel P (2020) Structural and socio-cultural barriers to accessing mental healthcare among Syrian refugees and asylum seekers in Switzerland. European journal of psychotraumatology 11(1), 1717825. 32128044 10.1080/20008198.2020.1717825 PMC7034440

[ref27] Kivimäki M , Batty GD , Pentti J , Shipley MJ , Sipilä PN , Nyberg ST , Suominen SB , Oksanen T , Stenholm S and Virtanen M (2020) Association between socioeconomic status and the development of mental and physical health conditions in adulthood: a multi-cohort study. The Lancet Public Health 5(3), e140–e149. 32007134 10.1016/S2468-2667(19)30248-8

[ref28] Koppe G , Meyer-Lindenberg A and Durstewitz D (2021) Deep learning for small and big data in psychiatry. Neuropsychopharmacology 46(1), 176–190. 32668442 10.1038/s41386-020-0767-z PMC7689428

[ref29] Lee SJ , Lawrence R , Bryce S , Ponsford J , Tan EJ and Rossell SL (2021) Emotional discomfort mediates the relationship between self-efficacy and subjective quality of life in people with schizophrenia. Journal of Mental Health 30(1), 20–26. 30879374 10.1080/09638237.2019.1581355

[ref30] Leichsenring F , Luyten P , Hilsenroth MJ , Abbass A , Barber JP , Keefe JR , Leweke F , Rabung S and Steinert C (2015) Psychodynamic therapy meets evidence-based medicine: a systematic review using updated criteria. The Lancet Psychiatry 2(7), 648–660. 26303562 10.1016/S2215-0366(15)00155-8

[ref31] Melo APS , Dippenaar IN , Johnson SC , Weaver ND , de Assis Acurcio F , Malta DC , Ribeiro ALP , Júnior AAG , Wool EE and Naghavi M (2022) All-cause and cause-specific mortality among people with severe mental illness in Brazil’s public health system, 2000-15: a retrospective study. The Lancet Psychiatry 9(10), 771–781. 35964638 10.1016/S2215-0366(22)00237-1 PMC9477749

[ref32] Miao Z , Wang Y and Sun Z (2020) The relationships between stress, mental disorders, and epigenetic regulation of BDNF. International journal of molecular sciences 21(4), 1375. 32085670 10.3390/ijms21041375 PMC7073021

[ref33] Nakao M , Shirotsuki K and Sugaya N (2021) Cognitive-behavioral therapy for management of mental health and stress-related disorders: recent advances in techniques and technologies. BioPsychoSocial medicine 15(1), 16. 34602086 10.1186/s13030-021-00219-w PMC8489050

[ref34] Pinto J , van Zeller M , Amorim P , Pimentel A , Dantas P , Eusébio E , Neves A , Pipa J , Clara ESanta , Santiago T , Viana P and Drummond M (2020) Sleep quality in times of Covid-19 pandemic. Sleep Medicine 74, 81–85. 32841849 10.1016/j.sleep.2020.07.012 PMC7366086

[ref35] Pranckutė R (2021) Web of Science (WoS) and Scopus: the titans of bibliographic information in today’s academic world. Publications 9(1), 12.

[ref36] Richards G and Stenner P (2022) Putting psychology in its place: Critical historical perspectives. London, UK: Routledge.

[ref37] Sainz RM , Mayo JC , Rodriguez C , Tan DX , Lopez-Burillo S and Reiter RJ (2003) Melatonin and cell death: differential actions on apoptosis in normal and cancer cells. Cellular and Molecular Life Sciences CMLS 60(7), 1407–1426. 12943228 10.1007/s00018-003-2319-1 PMC11138606

[ref38] Sameroff AJ and Seifer R (2021) Accumulation of environmental risk and child mental health. In Fitzgerald E, Lester BM, ZuckermanB (eds) Children of Poverty. New York, USA: Routledge.

[ref39] Scott AJ , Webb TL , Martyn-St James M , Rowse G and Weich S (2021) Improving sleep quality leads to better mental health: a meta-analysis of randomised controlled trials. Sleep Medicine Reviews 60, 101556. 34607184 10.1016/j.smrv.2021.101556 PMC8651630

[ref40] Shalaby RAH and Agyapong VIO (2020) Peer support in mental health: literature review. JMIR mental health 7(6), e15572. 32357127 10.2196/15572 PMC7312261

[ref41] Singh VK , Singh P , Karmakar M , Leta J and Mayr P (2021) The journal coverage of Web of Science, Scopus and dimensions: a comparative analysis. Scientometrics 126, 5113–5142.

[ref42] Smith JM , Lee AC , Zeleznik H , Scott JPCoffey , Fatima A , Needham DM and Ohtake PJ (2020) Home and community-based physical therapist management of adults with post-intensive care syndrome. Physical therapy 100(7), 1062–1073. 32280993 10.1093/ptj/pzaa059 PMC7188154

[ref43] Su C , Xu Z , Pathak J and Wang F (2020) Deep learning in mental health outcome research: a scoping review. Translational Psychiatry 10(1), 116. 32532967 10.1038/s41398-020-0780-3 PMC7293215

[ref44] Tsankova N , Renthal W , Kumar A and Nestler EJ (2007) Epigenetic regulation in psychiatric disorders. Nature Reviews Neuroscience 8(5), 355–367. 17453016 10.1038/nrn2132

[ref45] Vigo D , Jones L , Atun R and Thornicroft G (2022) The true global disease burden of mental illness: still elusive. The Lancet Psychiatry 9(2), 98–100. 35026138 10.1016/S2215-0366(22)00002-5

[ref46] Watts BV , Peltzman T and Shiner B (2021) Mortality after electroconvulsive therapy. The British Journal of Psychiatry 219(5), 588–593. 35048831 10.1192/bjp.2021.63

[ref47] Zare A , Bahia NJ , Eidy F , Adib N and Sedighe F (2019) The relationship between spiritual well-being, mental health, and quality of life in cancer patients receiving chemotherapy. Journal of Family Medicine and Primary Care 8(5), 1701–1705. 10.4103/jfmpc.jfmpc_131_19 PMC6559056 31198740

[ref48] Zhang J , Paksarian D , Lamers F , Hickie IB , He J and Merikangas KR (2017) Sleep patterns and mental health correlates in US adolescents. The Journal of Pediatrics 182, 137–143. 27939122 10.1016/j.jpeds.2016.11.007

